# A comparison of oral bacteriome isolated from periodontal pockets of participants with or without diabetes mellitus in Uganda: a case control study

**DOI:** 10.1186/s13104-024-06804-w

**Published:** 2024-05-22

**Authors:** Haruna Muhmood Kiryowa, William Buwembo, Ian Guyton Munabi, Erisa Sabakaki Mwaka, Charles Mugisha Rwenyonyi, Mark Kaddumukasa, Sarah Kiguli

**Affiliations:** 1 School of Biomedical Sciences, Makerere University College of Health Sciences, P.O. Box 7072, Kampala, Uganda; 2 School of Dentistry, Makerere University College of Health Sciences, P.O. Box 7072, Kampala, Uganda; 3 School of Medicine, Makerere University College of Health Sciences, P.O. Box 7072, Kampala, Uganda

**Keywords:** Diabetes mellitus, Microbiome, OTUs, Periodontal pockets and periodontitis

## Abstract

**Objective:**

Diabetes mellitus predisposes patients to increased incidence and severe forms of periodontal disease. Currently, information on the bacterial diversity of patients with diabetes mellitus and periodontitis in Uganda is scanty. This study set out to describe the bacteria associated with periodontitis in patients with diabetes mellitus in Uganda, as part of a larger study describing the association between periodontal disease and diabetes mellitus.

**Results:**

This was a case control involving 45 samples of gingival crevicular fluid collected from participants with periodontitis, the cases being 26 participants with diabetes mellitus and controls 19 participants without diabetes mellitus. Sequencing using the 16s Oxford nanopore long read protocol was followed by a bioinformatics analysis pipeline for alpha and beta diversity indices in the two groups. Multivariate tests were done to determine the differences in the bacterial composition in the two groups. Of the 739 Operational Taxonomic Units and 500 phyla identified, 37.9% (280/739) were from participants with diabetes mellitus. Analysis of beta diversity revealed a dissimilarity between the two study groups (CAP score = 0) with a significant association noted between periodontitis and the subgingival bacteria (*P* = 0.001). Diabetes mellitus reduced the quantity and altered the composition of the subgingival microbiome in the study participants.

## Introduction

Periodontitis is a chronic multifactorial inflammatory condition, associated with accumulation of dental plaque, that affects the supportive structures of the teeth leading to loss of attachment and subsequent tooth loss [1]. Other risk factors for periodontitis include old age, male sex, smoking, poor oral hygiene and low socioeconomic status [2–4]. Periodontitis has been described both as a local and systemic inflammatory condition. Apart from the local response to the oral bacteria, products of these microorganisms, notable amongst them is the Lipopolysaccharide (LPS), are potent immunomodulators and are responsible for the systemic inflammation observed in patients with severe periodontitis [5].

Therefore, the host’s immune response plays an important role in the progression of the disease as well as its association with systemic conditions [6]. Management of inflammation thus plays an important role in the control of bacteria as well as disease progression [7].

Diabetes mellitus is a group of metabolic diseases characterized by chronically elevated blood glucose levels as a result of defects in insulin secretion, insulin action or both [8]. It is classified into type 1 diabetes mellitus, type 2 diabetes mellitus, gestational diabetes mellitus and other types of unknown pathophysiology [9–12].

Diabetes mellitus is a known risk factor for periodontitis [13]. The prevalence rates of periodontitis have been reported to be twice as high in patients with diabetes mellitus compared with the non-diabetics [14, 15]. Diabetes mellitus leads to formation of endogenous activated glycation end products (AGEs) that are deposited in the different tissues including the periodontium [16, 17].

Periodontitis is a bacterial infection caused by gram negative anaerobes [12, 18]. These are also known as the red complex and include *Porphyromonas gingivalis*, *Tanerrella forsythus* and *Treponema denticola* [19–21]. Diabetes mellitus may alter the subgingival microbiome there by predisposing patients to a high prevalence and increased severity of periodontitis [22, 23].

Management of periodontal disease includes mechanical therapy, surgical treatment and use of antibiotics [24, 25]. Mechanical therapy, also known as scaling, root planning and polishing involves removal of plaque, a reservoir for bacteria that cause periodontal disease [26]. Surgical treatment is indicated in patients with exceptionally profound pockets or those where the conventional mechanical treatment has been unsuccessful. The choice of surgery depends on the severity of these but the ultimate aim is preventing progression of disease and regeneration of the lost tissues [27]. More advanced surgical techniques using dentinal derivatives have demonstrated strong regenerative properties on the periodontal tissues which may be helpful even in patients with diabetes mellitus [25]. Antibiotics when used with mechanical therapy may confer additional benefits [28]. The choice of antimicrobial agents requires an insight in the type of bacteria associated with periodontitis in the patients suffering from diabetes mellitus. Unfortunately, data on oral bacterial diversity in the Uganda diabetic population is scanty. This study set out to determine the effect of diabetes mellitus on the subgingival microbiome in adult patients suffering from periodontitis.

## Methods

### Study design and study setting

This was a matched case control study in the Medical Outpatients and dental departments of Kiruddu National Referral Hospital between February 2022 and January 2023.

### Sample size

The study involved 45 samples [29] of gingival crevicular fluid collected from pockets of patients with a diagnosis of periodontitis using the Community Periodontal Index (CPI) values of 3 and 4 [30]. Twenty-six samples were from participants with a confirmed diagnosis of diabetes mellitus [31] while nineteen samples were from periodontitis participants with no clinical diagnosis of diabetes mellitus. The Diabetic participants were selected by systematic sampling from a larger study determining the prevalence and factors associated with periodontal disease [32]. Participants who were pregnant and those on broad spectrum antibiotics were excluded from the study.

### Study procedure

#### Sample collection and DNA extraction

About 4 to 6 sterile paper points were inserted in the periodontal pockets for 30 s, removed and stored in 2mls cryo- vials containing 100 μl of DNA shield (Zymoresearch, CA, USA). The samples were then transported on ice to the Molecular Biology Laboratory of department of Anatomy, Makerere University College of Health Sciences. The laboratory is located within the histology laboratory on the ground and first floor of the department. DNA was extracted from these samples using the Quick-DNA Mini prep Plus Kit (Zymoresearch, CA, USA) as per the manufacturer’s instructions. It was then quantified by Nanodrop One (Themo Fisher Scientific, California, USA).

#### PCR amplification

The extracted DNA was then subjected to amplification in a Simpli Amp Thermocycler (Applied Biosystems, Waltham, MA, USA).

We evaluated one ribosomal marker of full length 16 S rRNA gene. The DNA containing the target bacteria was amplified to target the 16SrRNA gene using the 16–27 F (TTTCTGTTGGTGCTGATATTGCAGAGTTTGATCMTGGCTCAG) and 16 S-1492R (ACTTGCCTGTCGCTCTATCTTCGGTTACCTTGTTACGACTT) primer sets. All the primers contained the Oxford Nanopore tag which is an overhang that allows barcoding the sample during the second barcoding PCR. The mixture for the full length 16SrRNA gene (25μL total volume) contained 10ng of DNA template, 5X LongAmp Taq buffer, 0.3mM dNTPs, 0.4 μM of each primer and 0.5 units of LongAmp Taq DNA Polymerase (New England BioLabs). The PCR conditions were: denaturization of 30 s at 98^o^C followed by 35 cycles of 15 s at 98^o^C, 15 s at 51^o^C, 45 s at 72^o^C and a final step of 7 min at 72 ^o^C and then hold at 4^0^C. The amplicons were then run on a 2% gel stained with 1 μg/mL of ethidium bromide and viewed under U.V light in a viewer box (Vilber E-box, Vilber, Deutschland GmbH. Wielandstrasse 2, Germany).

#### Nanopore sequencing

Sequencing was carried out using the SQK-LSK114 sequencing kit as per manufacturer’s instructions as follows.

- DNA repair and end-prep for 35 min.
- Adapter ligation and clean-up for 30 min.
- Loading the Flonge flow cell.

After PCR, the amplicons were cleaned up with Clean NGS beads (Coenecoop 75, 2741 PH Waddinxveen, Netherlands) with the ratio of 1:2 of the amplicons to the beads as per the manufacturer’s instructions. A second bead clean of 1:1 was performed using the supernatant and the beads. The supernatant was removed the DNA bound to the beads was washed twice with 800ul of freshly prepared 70% ethanol. Without disturbing the beads. The beads and DNA were re suspended in 10 μl of Nuclease free water and incubated at room temperature for 10 min. The sample containing DNA and the beads was pelleted on a magnetic rack and the supernatant containing DNA was collected. The cleaned amplicons were quantified using a Qubit™ 4 Fluorometer (Themo Fisher Scientific, City, Singapore). The purified PCR products were used for Nanopore PCR barcoding using the EXP-PBC096 PCR barcoding expansion pack (Oxford Nanopore Technologies plc, Oxford, UK) under the following conditions 37^0^C for 20 min and 87^0^C for 20 min. The barcoded PCR products were purified with Clean NGS beads and then pulled with equal amounts for Ligation using the SQK-LSK114-XL kit (Oxford Nanopore Technologies plc, Oxford, UK) as per the manufacturer’s instructions. The eluted DNA library was quantified using a Nano drop One (Themo Scientific, USA). The quantified DNA library consisting of 800ng was sequenced using MinION MIN-101B platform on a flongle Flow Cell FLO-FLG114 (Oxford Nanopore Technologies plc, Oxford, UK) until a coverage of seven to ten times was achieved.

#### Base calling, bacteria identification and cleaning

The samples were then subjected to basecalling with a quality score of 9 over a 24-hour period. Only the reads that passed and were longer than 200 bases long were retained. These were aligned against the silva database 138 using minimap2 to identify the bacteria. A series of inhouse R, version 4.2.2, scripts were used to clean the data and create the Phyloseq objects which was used in the analysis of the data. Analysis involved recording the total number of OTUs followed by a series of comparisons using the Phyloseq [33] and Vegan [34] packages in R. Using a Phyloseq and Vegan packages, we tested for differences in alpha and phylogenetic diversity between the two groups. Analysis of Variance was used to determine the factors associated with the differences in microbial composition of the two groups.

### Descriptive characteristics

A total of 45 samples of gingival crevicular fluid were collected from 45 study participants. Twenty-six samples were from participants with diabetes mellitus and the nineteen samples were from participants without diabetes mellitus. The sociodemographic characteristics of the participants are summarized in Table 1.

**Table 1:** Sociodemographic characteristics of the study participants

Variable	Overall (*N* = 45)
**Gender**	
Female	26 (57.8%)
Male	19 (42.2%)
**Age in years**	
Mean (SD)	46.9 (13.5)
Median [Min, Max]	49.0 [23.0, 67.0]
**Level of education**	
No formal education	8 (17.8%)
Primary	20 (44.4%)
Secondary	13 (28.9%)
Tertiary	4 (8.9%)
**Diabetic medications**	
Both	8 (17.8%)
Insulin	3 (6.7%)
None	19 (42.2%)
Oral	15 (33.3%)
**Duration with Diabetes mellitus**	
None	19 (42.2%)
Less than 1 years	2 (4.4%)
2 to 5 years	8 (17.8%)
6 to 10 years	4 (8.9%)
greater than 10 years	12 (26.7%)
**Random blood sugar levels**	
Mean (SD)	9.44 (6.16)
Median [Min, Max]	7.80 [4.10, 33.8]
**Diabetic status**	
Diabetic	26 (57.8%)
Normal	19 (42.2%)

### Alpha diversity of the study participants

**Fig. 1:**
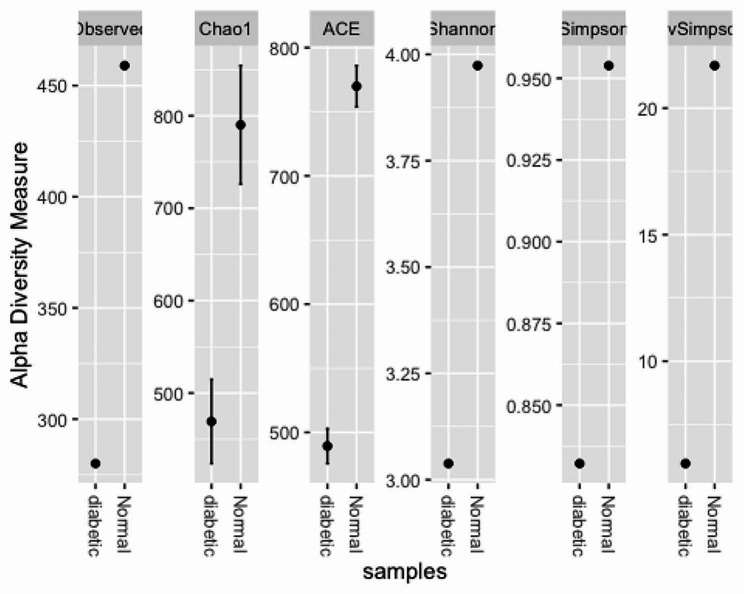
Alpha diversity measure plots with diabetic and non-diabetic groups in gingival crevicular fluid samples of participants with periodontitis

**Fig. 2:**
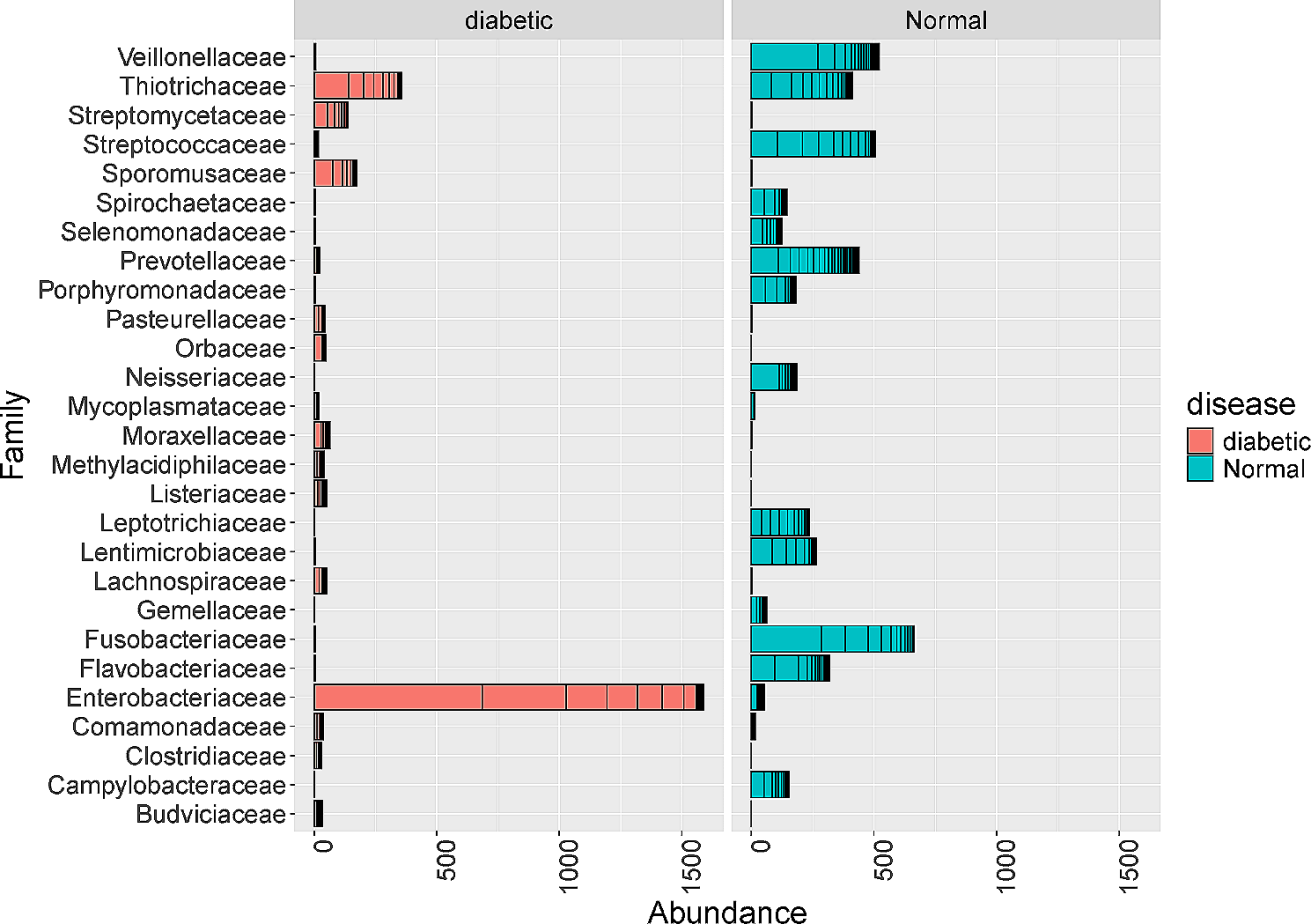
A figure illustrating the 30 most abundant and significant taxa in the two study groups

Out of the 739 Operational Taxonomic Units (OTUs), the participants with diabetes mellitus had 280 OTUs while those without diabetes mellitus had 459 OTUs. There was a marked difference in the taxa abundance between the two study groups (Fig. 1). The fifty most common families of bacteria are shown in Fig. 2.

### Beta diversity of the study participants

**Fig. 3:**
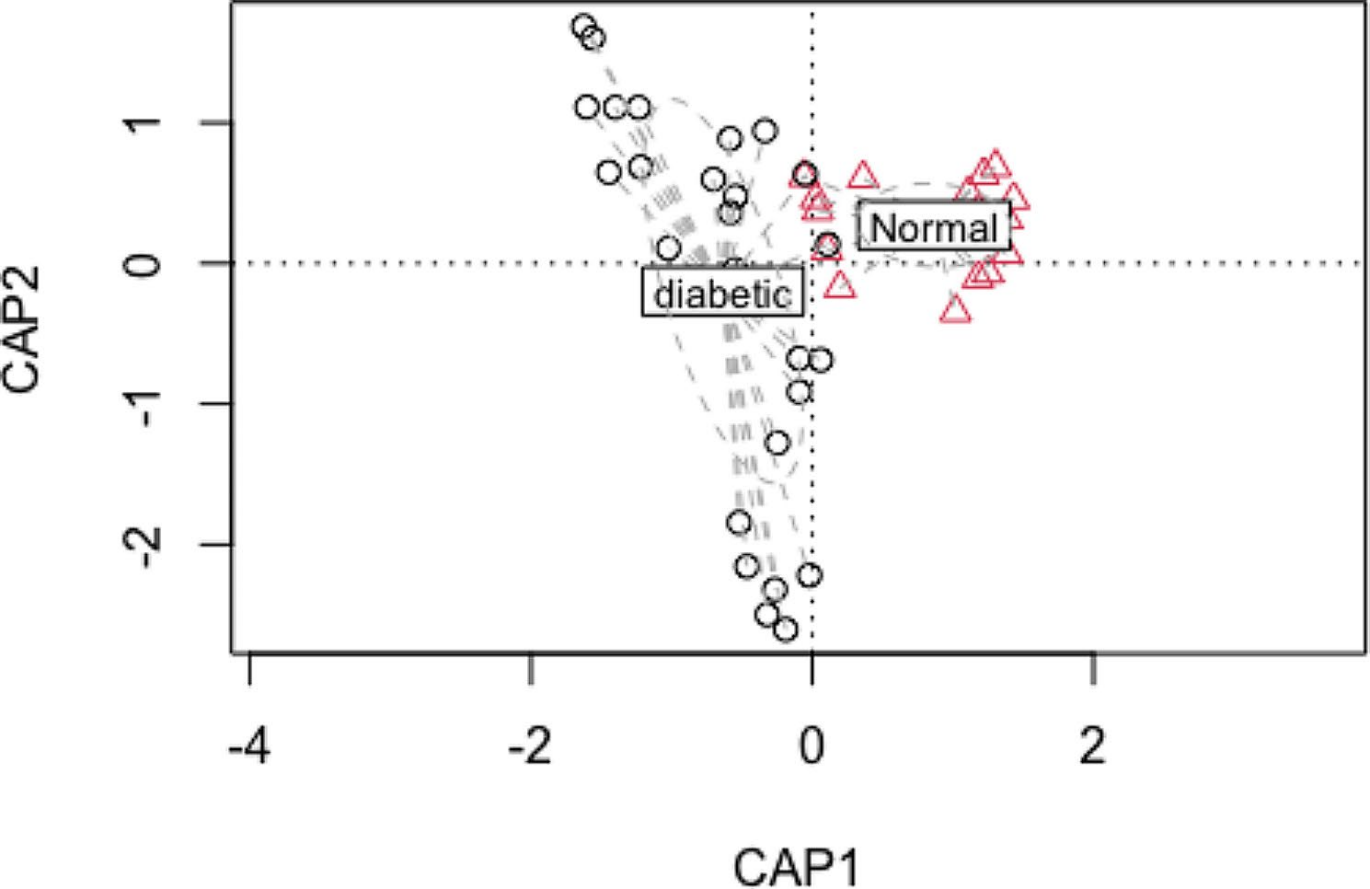
A graphic representation of constrained analysis of principle coordinates (CAP) illustrating the dissimilarity between the two study groups

There was a significant difference in microbiome between the participants with diabetes mellitus and those without diabetes mellitus. Participants with diabetes mellitus had a predorminancy of Enterobacteriaceae, Thiotrichaceae, Sporomucaceae and streptomycetaceae while those without diabetes mellitus had a predorminancy of Fusobacteriaceae, Veillonellaceae, Streptococcaceae and Prevotellaceae (Fig. 2). A constrained analysis of principle coordinates revealed a great dissimilarity between the two study groups (Fig. 3).

### Constrained analysis of principal coordinated of the factors associated with beta diversity

There was a significant association between periodontitis and beta diversity between the participants with diabetes mellitus and those without diabetes mellitus with *p* = 0.001 (Table 2).

**Table 2:** A table illustrating constrained analysis of principal coordinates (CAP) to test for significancy of selected explanatory variables

Attribute	DF	Sum of Sqs	F	Pr (> F)
Diabetic status	1	1.7971	4.4924	0.001 ***
Sex	1	0.2975	0.7436	0.833
Age	1	0.4098	1.0243	0.391
Level of education	3	1.1139	0.9282	0.644
Type of diabetic medications	2	0.9303	1.1627	0.194
Years with diabetes mellitus	3	1.3972	1.1642	0.163
Fasting blood sugar levels	1	0.2768	0.6920	0.893
Residual 32 12.8011				

## Discussion

This study set out to describe the bacteria associated with periodontitis in adults with diabetes mellitus in Uganda. It is the first of its kind in Uganda. This study identified over 500 families of bacteria in the subgingival fluid samples collected from the study participants. Generally, participants with diabetes mellitus had a marked decrease in the quantity of the subgingival microbiome compared to those without diabetes mellitus. Furthermore, there was a significant difference in the subgingival microbiome of participants with participants fewer OTUs than those without diabetes mellitus. The observed differences were significantly associated with periodontitis.

Our study reports a difference in alpha diversity in the study participants with participants suffering from diabetes mellitus contributing only 38% of the total OTUs (Fig. 1). Our findings are in line with Saeb et al. [35] who reported a reduced alpha diversity in participants with diabetes mellitus compared to those without diabetes mellitus. This study thus contradicts an earlier study by Taylor et al. [36] that refutes claims of diabetes mellitus affecting the subgingival microbiome citing lack of sufficient scientific evidence. A more recent study by Santos et al. [37] characterizing oral microbiome in patients with diabetes mellitus reports no difference in alpha and beta diversity between the non-diabetic and patients with diabetes mellitus. It should however be noted that that particular study used saliva samples while the current study used gingival crevicular fluid samples.

This study reports a difference in the oral microbiome composition between the two study groups which was significantly associated with the diabetic status.

Diabetes mellitus is characterized by chronically elevated blood sugar levels in blood. Gupta et al. [38] has reported a positive correlation between the patient’s glycemic state and the salivary glucose levels. Fermentation of these sugars releases acids hence favoring the survival of acidogenic and aciduric bacteria. Indeed, Cena et al. [39] has reported a higher abundancy of acid associated bacteria notably Lactobacillus, Tanerrella and Veillonela in diabetic patients compared to the normoglyceamic participants. Results from the current study show a lower abundancy of Veillonella in patients with diabetes mellitus compared to those without diabetes mellitus. Other acidogenic bacteria like Streptococcus and Prevotella were also reduced. This is further supported by the regression model which revealed no association between glycemic state and the beta diversity. However, one lactate forming bacteria, Sporomusaceae [40] was exclusively found in participants with diabetes mellitus.

The severity of periodontitis, clinically determined by the pocket depth may have an effect in the diversity of the microbiome. Deeper pockets are likely to favor survival of anaerobes than aerobes. Cai et al. [41] in their study comparing the oral microbiome in patients with periodontitis and those without periodontitis noted a significant increase of Spirochetes, Bacteroides and Fusobacteria. These bacteria are all obligate anaerobes. Results from the present study report a high prevalence of Moraxella and Listeria in participants with diabetes mellitus. These are aerobes that are more likely to survive in shallow pockets. There is a likelihood that participants with diabetes mellitus had mild forms of periodontitis compared to those who were normal. This is likely due to the fact that most of the participants without diabetes mellitus were selected from patients with periodontal disease attending the dental clinics while the cases were patients who had come to hospital for diabetic care and not periodontal treatment.

The role of medications in patients with diabetes mellitus may play a role in the variations observed in the oral microbiome. A part from the hypoglycemics, patients with diabetes mellitus are more like to be affected by a number of medical conditions that may necessitate the need for medications [42]. Metformin, a common oral hypoglycemic has been reported to exhibit an antibacterial effect when taken with other antibiotics [43]. Kizito et al. [44] in their study on antibacterial use in patients admitted on medical wards documented a wider prescription of broad-spectrum antibiotics. The greater use of these antibiotics may partly be responsible for the reduced microbial diversity observed in patients with diabetes mellitus.

### Limitations

The study did not consider antimicrobial resistance, which may undue influence the inference on use of antibiotics in the management of periodontitis in the studied population.

The study did not measure the sugar concentrations in saliva, which would provide information on the effect of salivary sugar levels on the oral microbiome.

Though the type of diabetic medication was recorded, the study participants were not classified into type 1 or type 2 DM. This may have affected the results obtained from this study.

This study was carried out in a low resource setting and may this not be representative of the larger diabetic population.

## Conclusion

The diabetic status significantly reduced the subgingival microbiome in patients with periodontitis. There is a need for further studies to strengthen the correlation of the oral microbiome isolated from periodontal pockets in subjects with or without diabetes in Uganda, highlighting the importance of managing periodontal health in diabetic patients and the appropriate measures to be taken to improve overall health outcomes and reduce the risk of health complications.

## Data Availability

The data is available at https://www.ncbi.nlm.nih.gov/sra/PRJNA1087153.

## Abbreviations

CAPs: Constrained Analysis of Principal Coordinates
DM: Diabetes Mellitus
DNA: Deoxyribose Nucleic Acid
MAKCHS-SBSREC: Makerere University College of Health Sciences School of Biomedical Sciences Research and Ethics Committee
OTUs: Operational Taxonomic Units
PCR: Polymerase Chain Reaction
rRNA: Ribosomal Ribose Nucleic Acid
